# Impact of Ebola and COVID-19 on maternal, neonatal, and child health care among populations affected by conflicts: a scoping review exploring demand and supply-side barriers and solutions

**DOI:** 10.1186/s13031-024-00572-x

**Published:** 2024-01-30

**Authors:** Yasir Shafiq, Elena Rubini, Zoha Zahid Fazal, Muhammad Murtaza Bukhari, Maheen Zakaria, Noor ul Huda Zeeshan, Ameer Muhammad, Luca Ragazzoni, Francesco Barone-Adesi, Martina Valente

**Affiliations:** 1 CRIMEDIM – Center for Research and Training in Disaster Medicine, Humanitarian Aid, and Global Health, Università del Piemonte Orientale, Novara, Italy; 2 Department of Translational Medicine, Università del Piemonte Orientale, Novara, Italy; 3 Centre of Excellence for Trauma and Emergencies (CETE) & Community Health Science, The Aga Khan University, Karachi, Pakistan; 4 Harvard Humanitarian Initiative, Department of Global Health and Population, Harvard T.H. Chan School of Public Health, Bostan, USA; 5 Department of Pediatrics, Brigham and Women’s Hospital, Global Advancement of Infants and Mothers, Boston, USA; 6 Medical College, Aga Khan University, Karachi, Pakistan; 7 VITAL Pakistan Trust, Karachi, Pakistan; 8 Department for Sustainable Development and Ecological Transition, Università del Piemonte Orientale, Vercelli, Italy

**Keywords:** Ebola, COVID-19, Maternal, Neonatal and child health, Primary health care

## Abstract

**Introduction:**

Armed conflicts have a severe impact on the health of women and children. Global health emergencies such as pandemics and disease outbreaks further exacerbate the challenges faced by vulnerable populations in accessing maternal, neonatal, and child healthcare (MNCH). There is a lack of evidence that summarizes the challenges faced by conflict-affected pregnant women, mothers, and children in accessing MNCH services during global health emergencies, mainly the Ebola and COVID-19 pandemics. This scoping review aimed to analyze studies evaluating and addressing barriers to accessing comprehensive MNCH services during Ebola and COVID-19 emergencies in populations affected by conflict.

**Methods:**

The search was conducted on PubMed, Scopus, and Web of Science databases using terms related to Ebola and COVID-19, conflicts, and MNCH. Original studies published between 1990 and 2022 were retrieved. Articles addressing the challenges in accessing MNCH-related services during pandemics in conflict-affected settings were included. Thematic analysis was performed to categorize the findings and identify barriers and solutions.

**Results:**

Twenty-nine studies met the inclusion criteria. Challenges were identified in various MNCH domains, including antenatal care, intrapartum care, postnatal care, vaccination, family planning, and the management of childhood illnesses. Ebola-related supply-side challenges mainly concerned accessibility issues, health workforce constraints, and the adoption of stringent protocols. COVID-19 has resulted in barriers related to access to care, challenges pertaining to the health workforce, and new service adoption. On the demand-side, Ebola- and COVID-19-related risks and apprehensions were the leading barriers in accessing MNCH care. Community constraints on utilizing services during Ebola were caused by a lack of trust and awareness. Demand-side challenges of COVID-19 included fear of disease, language barriers, and communication difficulties. Strategies such as partnerships, strengthening of health systems, service innovation, and community-based initiatives have been employed to overcome these barriers.

**Conclusion:**

Global health emergencies amplify the barriers to accessing MNCH services faced by conflict-affected populations. Cultural, linguistic, and supply-side factors are key challenges affecting various MNCH domains. Community-sensitive initiatives enhancing primary health care (PHC), mobile clinics, or outreach programs, and the integration of MNCH into PHC delivery should be implemented. Efforts should prioritize the well-being and empowerment of vulnerable populations. Addressing these barriers is crucial for achieving universal health coverage and the Sustainable Development Goals.

**Supplementary Information:**

The online version contains supplementary material available at 10.1186/s13031-024-00572-x.

## Introduction

Armed conflicts and persecutions have devastating consequences on the overall health and well-being of women and children, directly through violence as well as indirectly through various health effects, such as an increased risk of morbidity and mortality as well as the burden of undernutrition and infectious diseases [1]. Globally, more than 114 million people were displaced as a consequence of armed conflicts at the end of September 2023 [2]. More than 600 million women and girls are now living in countries affected by conflict, [3] nearly 50% more than what was recorded in 2017 [1, 3]. Women of reproductive age living in high-intensity conflict zones face a mortality risk that is three times higher than that of women living in peaceful settings [4, 5]. Further, 468 million children are forced to live in areas affected by armed conflict [6], these figures reaching the estimate of 36.5 million children displaced as a consequence of conflicts by the end of 2022 [7].

Global health emergencies, such as Ebola and COVID-19, amplify the strain on health systems, which, in turn, affects the overall health of populations [8]. Both Ebola and COVID-19 have starkly demonstrated this challenge [9, 10]. In the context of COVID-19, 90% of countries experienced disruptions to at least one essential health service, risking a rollback of nearly a decade of progress towards the United Nations 2030 Sustainable Development Goal (SDG) number 3 – Good Health and Well Being [11, 12]. Previously, during the Ebola epidemic, affected countries, primarily in Africa, witnessed a significant strain on their health systems, with many health facilities becoming overwhelmed or shutting down entirely [13]. In both low- and middle-income countries (LMICs) and high-income countries (HICs), such outbreaks often lead to the disruption or suspension of essential health services, resulting in reduced access to care [12, 14, 15]. This impact notably extends to MNCH services, especially at the primary health care (PHC) level, due to organizational and logistical challenges, such as staff and equipment shortages as well as broad public health restrictions [14]. Both epidemics saw many countries reporting a decline in antenatal care (ANC) consultations, decreased reliance on skilled birth attendance, and suboptimal vaccination rates [11, 16]. A recent study highlighted that while some critical health interventions like ANC and immunization are prioritized during conflicts, other essential MNCH services are not adequately provided in these settings [17]. The study also noted that the delivery and effectiveness of these interventions in conflict zones are largely influenced by international donors and highly dependent on the specific context of each conflict, with innovative solutions being developed to address these challenges [17].

The additional burden of pandemics and epidemics on MNCH services is usually difficult to quantify in conflict-affected countries due to huge constraints in accessing data and security challenges [18]. Therefore, the intersection of the MNCH, armed conflict, and pandemics is a critical area of study that deserves attention for several reasons. Conflict-affected populations, particularly women and children, already face numerous challenges in accessing healthcare due to displacement, economic instability, and disrupted health systems [19, 20]. Pandemics, such as Ebola and COVID-19, pose unique challenges to nearly all age groups [21–23] compared to endemic diseases (e.g., limited time and space) such as cholera, measles, malaria, or polio [16, 24]. Outbreaks induced by Ebola and COVID-19 viruses often suspend or disrupt essential health services, including MNCH. Beyond direct health consequences, these pandemics have broader societal impacts [23, 24], disrupting economies, social structures, and resource allocation [23, 24], with cascading effects on MNCH services. A previous review investigated interventions addressing infectious diseases affecting women and children in conflict-affected regions [25]. However, the authors focused solely on broader infectious diseases (e.g., malaria, polio, and tuberculosis) in conflict settings [25]. Another review delved into MNCH interventions within conflicts, thoroughly analyzing methodologies, barriers, and outcomes, without focusing on Ebola or COVID-19 [26]. A broader review examined MNCH interventions during pandemics and epidemics such as Zika, Ebola, and COVID-19 without including populations affected by conflict [16]. None of these reviews holistically assessed the intersection between MNCH, conflict, and global health emergencies, specifically pertaining to Ebola and COVID-19. The emergence of the Ebola and the COVID-19 pandemics has shown that many health systems face challenges in maintaining routine MNCH care because of the diversion of resources and staff to pandemic response efforts [16, 27]. Taken together, the intersection of vulnerabilities connected to different identities and statuses (e.g., being a refugee or internally displaced, a pregnant woman or a mother, or being a child mothered by them) may cause unique challenges to these populations in accessing MNCH services [28–30]. Data is needed to bridge the gaps towards continuity of MNCH care during pandemics among women and children affected by armed conflict [31, 32].

This scoping review aimed to thematically analyze original studies evaluating and addressing barriers to accessing MNCH services during Ebola and COVID-19 among conflict-affected pregnant women, mothers, and children under-five years of age. This will contribute to the assessment of barriers and potential solutions implemented or recommended in the published scientific literature. The findings aim to provide valuable insights for developing long-term strategies to enhance the quality of MNCH services for populations affected by conflict and with the additional burden of pandemics or epidemics.

## Methods

### Approach

A scoping review methodology was selected for its ability to map the breadth of research on MNCH in conflicts intersecting with Ebola and COVID-19 pandemics. This approach allows for the flexible incorporation of diverse studies, facilitating a comprehensive synthesis of the available evidence and the identification of significant research gaps. The scoping review's inherent adaptability makes it ideal for exploring complex, multifaceted public health challenges, where conventional systematic review protocols may be overly restrictive or inapplicable.

### Research question

This scoping review is centered around the research question: "What is the impact of Ebola and COVID-19 on MNCH care among populations affected by conflicts?" This question led to an exploration and synthesis of the available literature on the challenges, adaptations, and outcomes related to MNCH care in the context of these dual health emergencies in conflict-affected populations.

#### Search strategy and identification of relevant studies

A systematic search of original articles published from January 1, 1990, to August 7, 2023, was performed using three databases (PubMed, Scopus, and Web of Science). A manual search was conducted to identify other relevant studies. The timeframe was chosen because we aimed to retrieve evidence connected to Ebola and COVID-19, and existing or past conflicts (e.g., Afghan War, Iraq War, Syrian War, conflict in countries such as Ethiopia, Yemen, Sudan, Nigeria, Uganda, Sierra Leone, Guinea, and the Democratic Republic of Congo). Three concepts were used to develop the search syntax, encompassing: (a) Ebola and COVID-19, (b) conflict, and (c) MNCH. To broaden the search, the terms pandemic, epidemic, and disease outbreaks were also included. Irrelevant articles were excluded during screening (Additional file 1: Table S1).

#### Operational definitions

For operational purposes, the term “MNCH services” refers to services or care during pregnancy and childbirth at any level (community outreach, primary, secondary, and tertiary), postnatal care, newborn and under-five care, childhood immunization, nutrition, and family planning services including access to contraceptives. “Conflict” was employed as an umbrella term to encompass armed conflict, civil war, persecution, or ethnic violence [33]. In this paper the expressions “refugee women and children” and “women and children” refer to pregnant women or lactating mothers and their children under-five years of age living in conflict-affected settings or displaced because of hostilities who became refugees or internally displaced persons (IDP). Furthermore, this term also encompasses non-pregnant and non-lactating women with children under the age of five who require MNCH services. The term refugee is defined as a person who is outside their country of origin for reasons of feared persecution, conflict, or generalized violence that seriously disturbed public order and require international protection [34]. Similarly, IDP in the context of this review refers to those who had been forced to flee their homes or places of habitual residence, in particular as a result of or in order to avoid the effects of armed conflict or situations of generalized violence [35]. This review also encompasses countries that have experienced prolonged civil war or conflict, leading to economic and healthcare system fragility. The inclusion criteria for studies from these countries were established based on a window period of 15 years between the conflict and the period when data collection was reported. In this review, the terms “global health emergencies”, “pandemic”, “epidemic”, and “infectious disease outbreak” are employed interchangeably for Ebola and COVID-19, while the expression “barriers to access to care” will be used as an umbrella term to encompass the different elements presented in the conceptualization of Levesque et al. [36] We excluded the countries considered as “fragile” described in World Bank definition as “countries with high levels of institutional and social fragility, identified based on indicators that measure the quality of policy and institutions, and manifestations of fragility,” for example Venezuela, Zimbabwe etc [37].

### Study selection

Original studies focusing on MNCH services, assessing or reporting the challenges or barriers faced by the target population in accessing care, and describing interventions or strategies ensuring continuity of care were eligible for inclusion. Regarding global health emergencies, we only included articles addressing the effects of Ebola and COVID-19 viruses because these have emerged as health security threats globally or at least regionally and caused heightened disruption of health systems [27]. We included studies addressing the challenges affecting MNCH services in conflict-affected settings as per the operational definitions. Studies that targeted only health professionals or key informants involved in MNCH services were eligible for inclusion.

Articles that reported data from countries classified as “fragile” because of their high levels of institutional and social fragility were excluded. Emergencies related to climate-led or natural disasters were also excluded. Furthermore, studies targeting mental health and other PHC services that did not directly focus on MNCH were also excluded. Outbreaks related to measles, malaria, cholera, or polio were not eligible for inclusion since they did not have adverse consequences on health systems comparable to those induced by Ebola or COVID-19 [23]. Articles related to the Zika virus were excluded because most of the infections were in countries not affected by conflict [38]. Studies related to the Human Immunodeficiency Virus (HIV) pandemic were also excluded because the challenges faced by people infected by this etiological agent are distinct and protracted over a longer time span. As many refugees are resettled in HICs, no exclusion criteria were connected to research conducted in countries based on their income level [39]. The PRISMA guidelines were followed to report inclusion and exclusion criteria [40] (Additional file 2: Table S2: Eligibility criteria).

### Extraction and charting data

All identified indexed records were uploaded into Microsoft Excel version 2301 and duplicates were removed. Unique records from the databases were merged for screening. Four reviewers independently screened the titles and abstracts for relevance and any discrepancies were resolved through discussion or by a fifth reviewer. After the initial screening process, two reviewers assessed the full-text eligibility for inclusion. A comprehensive data extraction sheet was created to extract information for the thematic analysis. The data collection process included general information, variables such as setting and population characteristics, study design, objectives, and key findings. The results were categorized according to MNCH service domains. Furthermore, pertinent information was gathered if a study provided recommendations or described implemented strategies related to the continuity of care. To ensure accuracy, two reviewers entered the data, and any discrepancies or inconsistencies were resolved through discussion or with the assistance of a third reviewer. Data extracted from the studies were organized into a matrix that captured key information such as study location, methodology, population studied, main findings, and implications for MNCH care. This allowed for the systematic and thematic organization of the data, facilitating easier synthesis and analysis.

#### Data analysis, collating, summarizing, and reporting results

The findings from the selected studies were collated and summarized to provide a comprehensive overview of the impact of Ebola and COVID-19 on MNCH care to conflict-affected populations. We employed a narrative synthesis approach that enabled us to integrate the findings from diverse methodological backgrounds and draw broader conclusions about the overall trends and key issues identified in the literature. Descriptive statistics of the studies were performed to summarize the key characteristics, including geographic region, study type, type of conflict, displacement status of the population, and service characteristics (e.g., target population, MNCH service domain, and strategy applied). The MNCH domains or areas were categorized as community outreach, ANC, IPC, PNC, management of childhood illnesses, FP, vaccination, and nutrition. To report the findings, ANC and IPC were broadly defined as “Care during pregnancy and childbirth”, while PNC and under-five care, including vaccination and nutrition, were catalogued as “Preventive and curative newborn and childcare” and “FP.” A thematic analysis was conducted to report the impact of Ebola and COVID-19 on the continuity of care in MNCH services, and the strategies implemented or recommended in the studies. To facilitate the presentation of the findings related to health systems, the barriers and potential solutions were classified under the categories of “supply-side” and “demand-side” for all key domains of MNCH. The term “supply-side” is conceptualized as the various components related to the health system (e.g., service providers, service provision, supplies) [41], while “demand-side” encompasses individual behaviors, needs, and perceptions regarding services [41]. Additional file 3 provided reporting Items for scoping reviews (PRISMA-ScR) checklist.

## Results

### Characteristics of the included studies

**Fig. 1:**
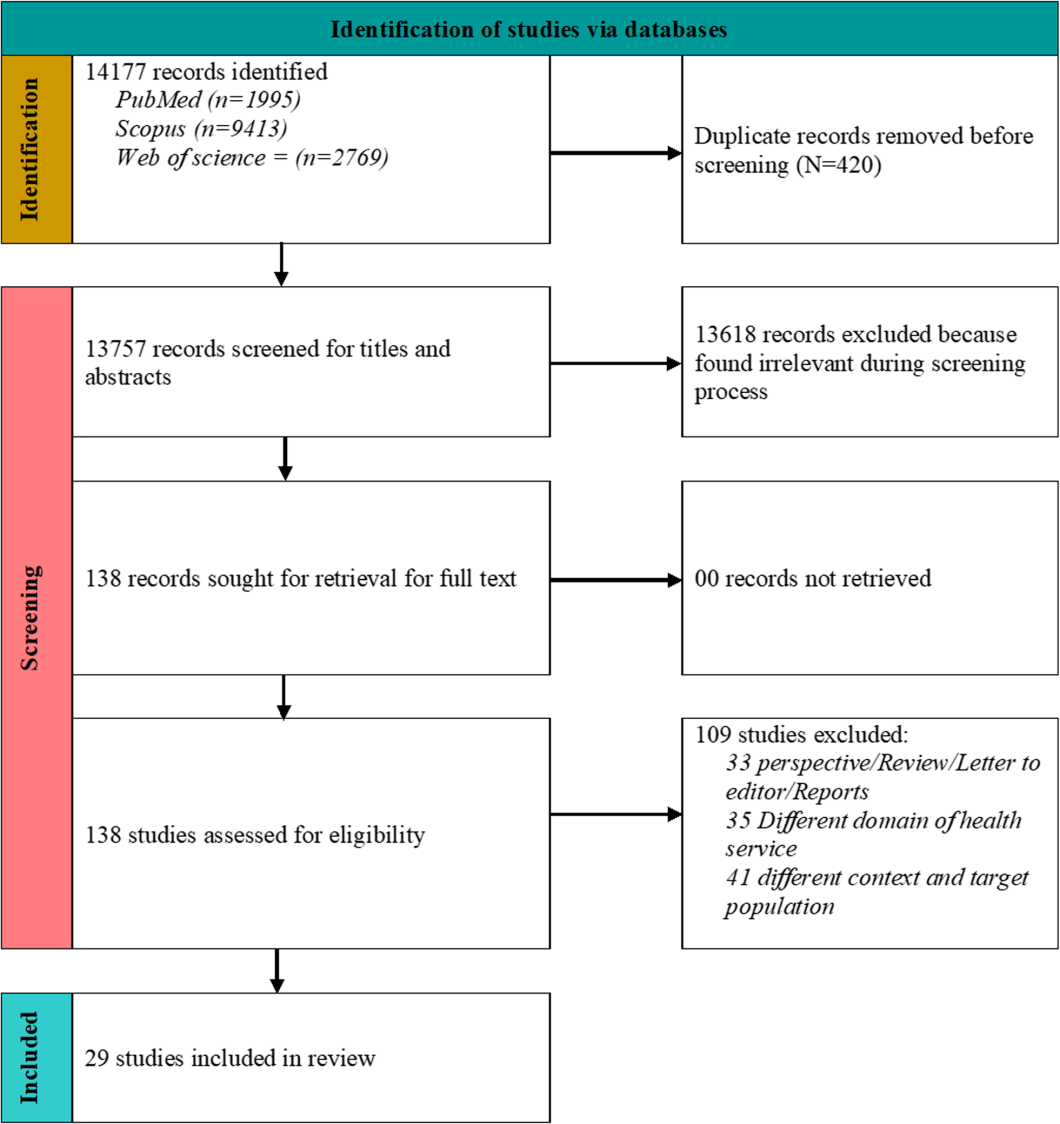
Prisma flow diagram. Providing details regarding the review screening and selection process

The search retrieved N = 13,757 studies, of which 29 met the eligibility criteria. The screening of sources and selection processes are shown in the PRISMA flow diagram (Fig. 1). A total of 13,618 records were excluded during screening because they were irrelevant and did not cover the scope. Of the 138 studies screened for full text, articles were excluded when they were non-original (N = 33), focused on a different domain of health services (N = 35), or when the context or target population did not match the inclusion criteria (N = 41).

Overall, the included studies were conducted in sub-Saharan and West Africa, South Asia, Middle East, and North America. The eligible studies adopted the following methodologies: mixed methods (N = 11, 37.9%), qualitative (N = 4, 13.7%), and quantitative (i.e., cross-sectional, or ecological survey and cohort studies (N = 14, 48.4%) (Table 1). The study participants were refugees (N = 9, 50%), population affected by a long civil war (14%), and population living in conflict or IDPs (N = 6, 10%). (Table 1).

**Table 1:** Description of the studies *Sources*: https://data2.unhcr.org/en/situations; https://www.worldbank.org/en/topic/fragilityconflictviolence/brief/harmonized-list-of-fragile-situations; https://www.cfr.org/global-conflict-tracker/conflict/violence-democratic-republic-congo

References	Title	Study design	Methods	Objectives	Study setting, study population status and status of conflict						Target population	MNCH service domain	Conclusions
					Setting where the study was conducted	Type of conflict	Year of conflict ^¶^	Duration from conflict to study of pandemic/ epidemic of interest (years)^¶^	Study population status	Type of pandemic or epidemic			
Altare et al. [43]	COVID-19 epidemiology and changes in health service utilization in Uganda’s refugee settlements during the first year of the pandemic	Secondary data analysis	Descriptive statistics, testing rates, incidence rates of COVID-19 cases, adjusted odds ratios for selected outcomes and applied interrupted time series analysis	To analyze the epidemiology of COVID-19 cases in Uganda’s refugee settlement, and evaluation of how health service utilization changed during the first year of the pandemic	Settlements in West Nile, South and Center regions of Uganda	Displaced population from multiple countries affected by conflicts: South Sudan, DR Congo, Somalia, Burundi, Rwanda	Conflict in multiple countries resulted in displacement	Not determined as study population is displaced	Refugees	COVID-19	Children	Postnatal care (PNC)	Routine and preventative health services appear to have been little affected by the COVID-19 pandemic, while immediate reductions were reported mostly for infectious disease consultations. The situation may have been very different in the second and third years of the pandemic, with more contagious variants
Barua et al. [47]	Community-based referral transportation system for accessing emergency obstetric services in the Rohingya refugee camp during the COVID-19 pandemic in Bangladesh: facilitators and barriers through beneficiaries’ and providers’ lens using a mixed-method design	Mixed methods	Survey among 100 women, the qualitative in-depth interviews with mothers and key informant interviews with providers	To present a community-based referral transportation system and explore its facilitators and barriers to improve the utilization of emergency obstetric services during the pandemic	Cox’s Bazar, Bangladesh	Persecution in Myanmar	2017	3	Refugees	COVID-19	Mothers	Intrapartum care (IPC)	Alliances and connections help reaching out to women who need emergency transport services and ensure access to the facility when needed
Rodo et al. [42]	A mixed methods study to assess the impact of COVID-19 on maternal, newborn, child health and nutrition in fragile and conflict-affected settings	Mixed methods	Key informant interviews	To investigate the collateral impacts of COVID-19 on funding, services and MNCHN outcomes in fragile and conflict affected setting (FCAs), as well as adaptations used in the field to continue activities	Afghanistan, DRC, Iraq, Somalia, Cameroon, South Sudan, Syria, Yemen, and Bangladesh	Fragile and conflict affected states, population in Conflict, and fragile settings. We only take findings which are collected from countries which are affected by conflict or hosting refugees because of conflict	Conflict in multiple countries resulted in displacement	Not determined	Refugees and internally displaced people (IDPs), Population affected by long civil war	COVID-19	Mothers and children	General MNCH, IPC, Antenatal care (ANC), Family planning (FP), PNC	Humanitarian actors have made several adaptations to continue providing MNCHN services during the pandemic; these strategies have often been implemented unevenly within and across settings, and not been evaluated
Njoh et al. [70]	Impact of periodic intensification of routine immunization within an armed conflict setting and COVID-19 outbreak in Cameroon in 2020	Cross-sectional	Survey	To assess the impact of periodic intensification of routine immunization (PIRI) on vaccination coverage and disease surveillance in the region	Southwest Region (SW) of Cameroon	Anglophone Crisis	2017	2	Population living in conflict or IDPs	COVID- 1	Children	PNC	PIRI improved the performance of routine vaccination coverage and disease surveillance of vendor drug programs (VPDs) in the SW of Cameroon in the context of insecurity and COVID-19. PIRI also helped to rapidly stimulate the uptake of newly introduced vaccines like the ones covering measles and rubella (MR-2) and human papilloma virus (HPV)
Hirani et al. [44]	Impact of COVID-19 on women who are refugees and mothering: a critical ethnographic study	Qualitative	Critical ethnographic study. Field observations, review of media reports, and in-depth, semi-structured interviews with study participants	To explore the impact of COVID-19 on women who are refugees and mothering young children aged 2 and under in Saskatchewan, Canada, as well as to explore major barriers (sociocultural, environmental, and economic) and determinants causing stress and adding to the vulnerability of women during the COVID-9 pandemic	Saskatchewan community, Canada	Displaced population from multiple countries affected by conflicts: Middle East and Africa	Conflict in multiple countries resulted in displacement	Not determined	Refugees	COVID-19	Mothers and Children	ANC, IPC, PNC	Refugee women with young children are at risk of experiencing reduced physical, mental, and emotional well-being. During COVID-19, women who are refugees and mothering are at high risk of experiencing add on stressors due to limited social support, difficulty accessing health care, and other COVID-19-related restrictions put in place in their social environment. Fear of getting sick, limited socialization, lack of social support, economic difficulties, limited follow-up community-based care, inability to access health-care settings, and restrictions on their ability to stay with their sick hospitalized child due to COVID-19 restrictions caused negative effects on mothering refugees' mental health
Barua et al. [55]	Implementation of a community-based referral project to improve access to emergency obstetric and newborn care in Rohingya population during COVID-19 pandemic in Bangladesh	Mixed methods	Secondary data of routine utilization of the 12 referral hubs through key informant interviews and a community survey conducted with 100 pregnant women	To describe the implementation process of the Referral Hub (RH) and present clients’ utilization and perception of the service	Refugee camps Bangladesh	Persecution in Myanmar	2017	5	Refugees	COVID-19	Mothers	ANC	The RH is a timely innovation to increase access to emergency obstetric care in the Rohingya population even during the COVID-19 pandemic. Moreover, it is a boon to the Rohingya community that otherwise lacks proper and easy access to transport facilities, especially during an emergency. The success is evident from the increasing utilization and recommendations from clients
Stirling et al [45]	“COVID affected us all:” the birth and postnatal health experiences of resettled Syrian refugee women during COVID-19 in Canada	Qualitative	Individual, semi-structured interviews	To understand the experiences of resettled Syrian refugee women accessing PNC and social support	Nova Scotia, Canada	Syrian war	2011	8	Refugees	COVID-19	Mothers and Children	IPC, PNC, FP	Equity-oriented approach must be taken to reduce reproductive health disparities for resettled refugee women
Lusambili et al. [52]	“We have a lot of home deliveries” A qualitative study on the impact of COVID-19 on access to and utilization of reproductive, maternal, newborn and child health care among refugee women in urban Eastleigh, Kenya	Qualitative	In-depth interviews	To improve understanding of the impact of COVID-19 on women refugees' access to and utilization of antenatal care, delivery and PNC in Eastleigh, Kenya in order to identify existing gaps and inform potential interventions that could improve uptake of services during the COVID-19 pandemic	Eastleigh, Kenya	Displaced population from multiple countries affected by conflicts: Somalia, Ethiopia, Tanzania, Uganda, Eritrea, and South Sudan. Most of the refugees are of Somali	Conflict in multiple countries resulted in displacement	Not determined	Refugees	COVID-19	Mothers and Children	ANC, IPC, PNC	Findings identify gaps in existing national policies and call for urgent consideration for refugee women who have no access to facility-based skilled care during a pandemic. Findings show that refugees delayed uptake of RMNCH care, and facilities reported low attendance. This was often a result of refugees’ fear of contracting COVID-19 together with poverty, which meant that they could not afford masks or the cost of private maternity services
Altare et al. [46]	COVID-19 epidemiology and changes in health service utilization in Azraq and Zaatari refugee camps in Jordan: A retrospective cohort study	Observational	Secondary data analysis	To describe the epidemiology of COVID-19 in Azraq and Zaatari refugee camps in Jordan and evaluate changes in routine health services during the COVID-19 pandemic	Azraq and Zaatari refugee camps, Jordan	Syrian war	2011	9	Refugees	COVID-19	Mothers and Children	ANC, FP, PNC	The pandemic has both exacerbated existing inequalities and demonstrated that until all populations are included in national response plans, the world remains vulnerable to the current and the next pandemic
Hossain et al. [66]	Exploring healthcare-seeking behavior of most vulnerable groups amid the COVID-19 pandemic in the humanitarian context in Cox’s Bazar, Bangladesh: Findings from an exploratory qualitative study	Mixed-method research	In-depth interviews	To understand the factors influencing healthcare-seeking behavior of the most vulnerable groups during COVID-19 pandemic	Ukhiya sub-district of Cox’s Bazar, Bangladesh	Persecution in Myanmar	2017	3	Refugees	COVID-19	Pregnant and lactating women as well as other population	ANC and IPC	The healthcare-seeking behavior of MVGs amid the COVID-19 pandemic in the context of Rohingya and the host communities of Cox’s Bazar was influenced by several factors ranging from socioeconomic, demographic, individual, health belief-related, and institutional factors. However, these factors are not linear rather they are intertwined, and their intersectionality represents diverse nuances of the lived realities of these most vulnerable groups during the COVID-19 pandemic
Galle et al. [49]	Utilization of services along the continuum of maternal healthcare during the COVID-19 pandemic in Lubumbashi, DRC: findings from a cross-sectional household survey of women	Cross-sectional	Survey	The continuum of maternal care along antenatal (ANC), intrapartum and postnatal care (PNC) is fundamental for protecting women’s and newborns’ health. The COVID-19 pandemic interrupted the provision and use of these essential services globally. This study examines maternal healthcare utilisation along the continuum during the COVID-19 pandemic in the Democratic Republic of the Congo (DRC)	Lubumbashi, DRC,	Armed conflict	2020 and later	1	Population living in conflict or IDPs	COVID-19	Pregnant women	ANC, intrapartum, and PNC	During the COVID-19 pandemic, maternal healthcare seeking behaviors were shaped by vaccine hesitancy and care unaffordability in Lubumbashi. Addressing the high cost of maternal healthcare and vaccine hesitancy appear essential to improve access to maternal healthcare
Nomhwange et al. [48]	Measles outbreak response immunization during the COVID-19 pandemic: lessons from Borno State, Nigeria	Retrospective	review assessment of the WHO framework, epidemiological reports and vaccination response data	documents the implementation of an outbreak response immunization (ORI) during the COVID-19 pandemic and the implementation of global guidelines for mass vaccination	Borno state across six local government areas (LGAs)	Armed conflict	2019	2	Population living in conflict or IDPs	COVID-19	Children	Vaccination	the WHO decision-making framework for implementing mass vaccinations in the context of the COVID-19 Pandemic was utilized for the outbreak response immunization in Borno State, Nigeria with 181,634 children aged 9 Months-9 years vaccinated with the measles vaccine. The use of the WHO decision-making framework to assess risk benefits of initiating mass vaccination campaigns remains a very important practical tool. These types of responses in Nigeria and other low- and middle-income countries (LMICs), with hitherto suboptimal immunization coverage and weak health systems and other settings, affected by humanitarian emergencies is essential in the achievement of the regional measle's elimination targets
Gizelis et al. [50]	Maternal Health Care in the Time of Ebola: A Mixed-Method Exploration of the Impact of the Epidemic on Delivery Services in Monrovia	Mixed methods	Multinomial logit model with in-depth semi-structured interviews	data on the utilization of maternal health care services from two representative surveys	Liberian DHS and urban Monrovia	Civil war	Ended in 2003	12	Population affected by long civil war	Ebola	Women	MNCH and Delivery services	Our findings indicate that resources to shore up healthcare institutions should be directed toward interventions that support private facilities and health personnel working privately in communities during times of crisis so that these facilities are safe alternatives for women during crisis
Elston et al. [51]	Maternal health after Ebola: unmet needs and barriers to healthcare in rural Sierra Leone	Mixed methods	Household survey targeting women who had given birth since onset of the Ebola outbreak; structured interviews at rural sites investigating maternal deaths and reporting; and in-depth interviews (IDIs) targeting mothers, community leaders and health workers	described health outcomes and health-seeking behavior amongst pregnant women to inform health policy	urban and rural areas of Tonkolili District of Seirra Leone	Civil war	Ended in 2002	12	Population affected by long civil war	Ebola	Postpartum mothers, community leaders and health workers	health-seeking behaviors, barriers to healthcare, and childbirth outcomes	Pregnant women faced important barriers to care, particularly in rural areas, leading to high preventable mortality and morbidity. Women wanted to access healthcare, but services available were often costly, unreachable and poor quality. We recommend urgent interventions, including health promotion, free healthcare access and strengthening rural services to address barriers to maternal healthcare
Delamou et al. [58]	Maternal and Child Health Services in the Context of the Ebola Virus Disease: Health Care Workers' Knowledge, Attitudes and Practices in Rural Guinea	Cross-sectional	standardized self-administered questionnaire	to document maternal and child health care workers' knowledge, attitudes and practices on service delivery before, during and after the 2014 EVD outbreak	ten health districts in rural Guinea	Armed conflict	Ended in 2001	14	Population affected by long civil war	Ebola	maternal and child health care workers	MNCH service delivery	Infection prevention and control measures established during the EVD outbreak have substantially improved self-reported provider practices for maternal and child health services in rural Guinea. However, more efforts are needed to maintain and sustain the gain achieved
Elston et al. [56]	Impact of the Ebola outbreak on health systems and population health in Sierra Leone	Mixed methods	interviews, focus groups, and interrogation and analysis of data from health facilities, district health records and burial teams	identify and quantify the impact of the Ebola outbreak on population health and health systems	Two districts of Sierra Leone	Civil war	Ended in 2002	12	Population affected by long civil war	Ebola	key local stakeholders, Ebola response team members; civil and traditional authority figures, HCWs, community workers, social mobilizers, patients, and NGO members	General MNCH service utilization	The findings indicate a public health emergency as a legacy of the Ebola outbreak. Sustained commitment of the international community is required to support health system re-building
Kotiso et al. [65]	Impact of the COVID-19 pandemic on the utilization of health services at public hospitals in Yemen: a retrospective comparative study	Retrospective	routinely hospital services data and medical records by using the DHIS2 system and by phone	Delivery of health services is investigated both before and during the outbreak of the COVID-19 pandemic at public hospitals in Yemen to assess the impact of COVID-19 on the utilization of health services	127 hospitals in Yemen	War in Yemen	2014	5	Population living in conflict or IDPs	COVID-19	General hospitals, maternal and children’s hospitals, psychiatric hospitals and district hospitals	continuity of MNCH services delivery	The impact of COVID-19 on continuity of health services delivery in Yemen has been distinct and profound, where the study revealed that the number of the consultations, surgeries and number of vaccinated children have been declined during the COVID-19 pandemic, likely due to the partially lockdown measures taken and fear of being infected. However, the deliveries and C-section services remained nearly in the same level and did not affect by the COVID-19 pandemic
Hategeka et al. [67]	Impact of the COVID-19 pandemic and response on the utilization of health services in public facilities during the first wave in Kinshasa, the Democratic Republic of the Congo	Observational	Monthly time series data from the DRC Health Management Information System (January 2018 to December 2020) and interrupted time series with mixed effects segmented Poisson regression models	Evaluated the impact of the pandemic on the use of essential health services (outpatient visits, maternal health, vaccinations, visits for common infectious diseases and non-communicable diseases) during the first wave of the pandemic in Kinshasa	Gombe commune of Kinshasa, city in Africa	Armed conflict	2020 and later	1	Population living in conflict or IDPs	COVID-19	health facilities (i.e., health centers and hospitals)	Health service use (outpatient visits, maternal health, vaccinations, visits for common infectious diseases and non-communicable diseases)	The COVID-19 pandemic resulted in important reductions in health service utilization in Kinshasa, particularly Gombe. Lifting of lockdown led to a rebound in the level of health service use but it remained lower than prepandemic levels
Quaglio et al. [61]	Impact of Ebola outbreak on reproductive health services in a rural district of Sierra Leone: a prospective observational study	Prospective Observational	MCH services uptake using routinely collected health services data	To assess the trends concerning utilization of maternal and child health (MCH) services before, during and after the Ebola outbreak, quantifying the contribution of a reorganized referral system (RS)	Pujehun district in Sierra Leone, 77 community health facilities and 1 hospital from 2012 to 2017	Civil war	Ended in 2002	12	Population affected by long civil war	Ebola	77 community health facilities and 1 hospital	Utilization of maternal and child health (MCH) services, institutional deliveries, Cesarean-sections, pediatric and maternity admissions and deaths, and major direct obstetric complications (MDOCs), at hospital level; (2) antenatal care (ANC) 1 and 4, institutional delivery and family planning, at community level	A stronger health system compared with other districts in Sierra Leone and a strengthened RS enabled health facilities in Pujehun to maintain service provision and uptake during and after the Ebola epidemic
McKay et al. [69]	Family Planning in the Sierra Leone Ebola Outbreak: Women's Proximal and Distal Reasoning	Qualitative	In-depth interviews	to explore women's perspectives on delaying pregnancy during the Ebola outbreak using family planning methods	Kambia District of West Africa	Civil war	Ended in 2002	12	Population affected by long civil war	Ebola	Women who were either family planning users or nonusers	FP use during outbreak	Using the lens of family planning to consider how women choose to access health care in an outbreak gives us a unique perspective into how all health care interactions are impacted by a generalized outbreak of Ebola, and how outbreak responses struggle to ensure such services remain a priority
Jones et al. [63]	'Even when you are afraid, you stay': Provision of maternity care during the Ebola virus epidemic: A qualitative study	A hermeneutic phenomenological approach	Face to face interviews	To explore nurse-midwives understanding of their role in and ability to continue to provide routine and emergency maternity services during the time of the Ebola virus disease epidemic in Sierra Leone	14 districts of Sierra Leone	Civil war	Ended in 2002	12	Population affected by long civil war	Ebola	Nurses, midwives, medical staff and managers providing maternal and newborn care during the Ebola epidemic in facilities designated to provide basic or emergency obstetric care	Overall MNCH	Nurse-midwives faced increased risks of catching Ebola compared to other health workers but continued to provide essential maternity care
Siekmans et al. [53]	Community-based health care is an essential component of a resilient health system: evidence from Ebola outbreak in Liberia	Mixed methods	collect data from CHWs (structured survey, n = 60; focus group discussions, n = 16), government health facility workers and project staff. Monthly data on child diarrhea and pneumonia treatment were gathered from CHW case registers and local health facility records	to examine the value of a community-based health system in ensuring continued treatment of child illnesses during the outbreak and the role that CHWs had in Ebola prevention activities	Bomi County, Montserrado County, Gbarpolu County	Civil war	Ended in 2003	12	Population affected by long civil war	Ebola	CHWs, government health facility workers and project staff	community-based treatment of child diarrhea and pneumonia	Investments in community-based health service delivery contributed to continued access to lifesaving treatment for child pneumonia and diarrhea during the Ebola outbreak, making communities more resilient when facility-based health services were impacted by the crisis. To maximize the effectiveness of these interventions during a crisis, proactive training of CHWs in infection prevention and “no touch” iCCM guidelines, strengthening drug supply chain management and finding alternative ways to provide supportive supervision when movements are restricted are recommended
Jones et al. [60]	‘Women and babies are dying but not of Ebola’: The effect of the Ebola virus epidemic on the availability, uptake and outcomes of maternal and newborn health services in Sierra Leone	Mixed methods	The number of antenatal and postnatal visits, institutional births, availability of emergency obstetric care (EmOC), maternal deaths and stillbirths were assessed by month, by districts and by level of healthcare for 10 months during, and12 months prior to, the Ebola virus disease (EVD)epidemic	to determine the impact of the Ebola virus epidemic on the availability, uptake and outcome of routine maternity services in SierraLeone	All healthcare facilities designated to provide comprehensive (n = 13) or basic (n = 67) EmOC across the 13 districts of Sierra Leone were included	Civil war	Ended in 2002	12	Population affected by long civil war	Ebola	Healthcare facilities designated to provide comprehensive (n = 13) or basic (n = 67) EmOC	Number of antenatal and postnatal visits, institutional births, availability of emergency obstetric care (EmOC), maternal deaths and stillbirths	During the EVD epidemic, fewer pregnant women accessed healthcare. For those who did, an increase in maternal mortality and stillbirth was observed. In the post-Ebola phase, ‘readiness’ (or not) of the global partners for large-scale epidemics has been the focus of debate. The level of functioning of the health system with regard to ability to continue to provide high-quality effective routine care needs more attention
Evens et al. [57]	"Africans, we know how to adapt indeed": Adaptations to family planning and reproductive health services in humanitarian settings in Nigeria during the COVID-19 pandemic	Mixed methods	quantitative analysis of data from routine programmatic activities, qualitative data from in-depth interviews (IDIs) with project staff and process documentation of programmatic activities and modifications	1) identify modifications in FP/RH services due to COVID-19, 2) understand staff perception of their utility and impact, and 3) gauge trends in key FP/RH in-service delivery indicators to assess changes prior to and after the March 2020 lockdown	Borno State and Cross River State, in Nigeria	Armed conflict	2019	2	Population living in conflict or IDPs	COVID-19	data from routine programmatic activities, project staff and process documentation of programmatic activities and modifications	Family Planning/Reproductive Health (FP/RH) services	Lessons learned included the need to better sensitize and educate communities, maintain FP commodities and increase support provided to health workers. Deliberate adaptations in IHANN II and UNHCR-SS-HNIR projects turned challenges to opportunities, ensuring continuity of services to the most vulnerable populations
Tesfai [64]	Human Rights Violations and Mistrust among Refugees in South Africa: Implications for Public Health during the COVID Pandemic	Qualitative	In-depth interviews	about refugees’ access to healthcare in South Africa during the COVID-19 pandemic and the consequences of inconsistent access and discrimination on their trust of public healthcare initiatives	South Africa	Armed conflict	Conflict in multiple countries resulted in displacement	Not determined as study population is displaced	Refugees	COVID-19	11 key stakeholders from the refugee community, 7 community leaders, and 4 NGO staff members who served refugee communities	refugees’ access to healthcare in South Africa during the COVID-19 pandemic and the consequences of inconsistent access and discrimination on their trust of public healthcare initiatives	The results suggest that refugees’ access to public healthcare services were perceived as exclusionary and discriminatory. Furthermore, the growing mistrust in institutions and authorities, particularly the healthcare system, and misperceptions of COVID-19 compromised refugees’ trust and adherence to public health initiatives. This ultimately exacerbates the vulnerability of the refugee community, as well as the wellbeing of the overall population
Camara et al. [68]	Effect of the 2014/2015 Ebola outbreak on reproductive health services in a rural district of Guinea: an ecological study	Ecological study	routine service data	compared trends in family planning, antenatal care, and institutional deliveries over the period before, during and after the outbreak	Macenta district of Guinea	Armed conflict	2001	14	Population affected by long civil war	Ebola	all the health facilities data in Macenta district	trends in family planning, antenatal care, and institutional deliveries	All services assessed were affected by Ebola. Family planning recovered post-Ebola; however, shortfalls were observed in recovery of antenatal care and institutional deliveries. We call for stronger political will, international support and generous funding to change the current state of affairs
Shannon, II et al. [59]	Effects of the 2014 Ebola outbreak on antenatal care and delivery outcomes in Liberia: a nationwide analysis	Cross-sectional	Routinely reported program data	To determine access to antenatal care (ANC), deliveries and their outcomes before, during and after the 2014–2015 Ebola outbreak	All health facilities, public and private, in Liberia, West Africa	Civil war	Ended in 2003	12	Population affected by long civil war	Ebola	Women seeking ANC at health facilities, all institutional and community deliveries and all newborns	antenatal care (ANC) and deliveries	The Liberian health system was considerably weakened during the Ebola outbreak and had difficulties providing basic maternal health services. In the light of the major reporting gaps during the Ebola period, and the reduced use of health facilities for maternal care, these findings highlight the need for measures to avoid such disruptions during future outbreaks
Quaglio et al. [54]	Maintaining maternal and child health services during the Ebola outbreak: experience from Pujehun, Sierra Leone	Mixed methods	Hospital registers and contact tracing form data with healthcare workers and local population interviews, the transmission chain was reconstructed. Data on the utilization of maternal and neonatal health services were collected from the local district’s Health Management Information System	to provide information on understanding of how Ebola impacted maternal and child health services in Sierra Leone	Pujehun district, in Sierra Leone	Civil war	Ended in 2002	12	Population affected by long civil war	Ebola	Hospital registers and contact tracing form data with healthcare workers and local population	Women seeking ANC at health facilities, all institutional and community deliveries and all newborns	The Ebola outbreak reduced the number of patients at hospital level in Pujehun district. However, the activities undertaken to manage Ebola reduced the spread of infection and the impact of the disease in mothers and children. A number of reasons which may explain these results are presented and discussed
Caulker et al. [62]	Life goes on: the resilience of maternal primary care during the Ebola outbreak in rural Sierra Leone	Cross-sectional	secondary program data	To compare trends in antenatal care (the first and fourth visit [ANC1 and ANC4]), delivery, and postnatal care (PNC1) service utilization before, during and after the Ebola outbreak (2014–2016)	All 100 health facilities providing maternal services in Moyamba, Sierra Leone, a rural district that experienced a smaller Ebola outbreak than other areas	Civil war	Ended in 2002	12	Population affected by long civil war	Ebola	100 health facilities secondary program data	antenatal care (the first and fourth visit [ANC1 and ANC4]), delivery, and postnatal care (PNC1) service utilization	In a rural district less affected by Ebola transmission than other areas, utilization of maternal primary care remained robust, despite the outbreak

^¶^Authors of this scoping review derived this information from UNHCR, WORLD Bank data and other online information available from countries specific data as well as information provided in the articles

### Challenges and strategies

Our review examined the factors that affect continuity of care in MNCH services and explored potential strategies for addressing them. In this section, the supply- and demand-side challenges, and strategies to overcome them are described and analyzed.

### Challenges affecting continuity of care at supply-side

#### Accessibility barriers

‘Preventive and curative newborn and child care’ was highly impacted by ‘accessibility’ issues during the Ebola and COVID-19 [42–46]. In LMICs, geographical inaccessibility (Table 2), disruption of services, lack of services or supplies, or suspension of services were common and resulted in limited access to these essential health services [42, 43, 47]. Furthermore, some level of discrimination was also reported during the COVID-19 pandemic among refugee women, as mentioned by a social worker: *“Refugees have been experiencing the discrimination from many years in all departments …. They be afraid always triggered by their past experiences… which means there is no good experience someone is expecting [from] healthcare services.”* [61]

**Table 2:** Barriers to access to care in MNCH service domains by income level of the country and adopted solutions

Demand-side		MNCH domain	Supply-side	
Solutions	Barriers		Barriers	Solutions
Community engagement^ȸ^ Messaging system ^ȸ^	Diminished trust^¶ ȸ^	Community outreach	Disruption of services ^¶ ȸ^ Inadequate/limited personnel ^¶ ȸ^ Financial restrictions ^ɸ ȸ^ Redirection of resources ^ɸ ȸ^ Community uninvolved^¶c^ Limited access to community support ^¶ c^ Measures to deter gathering^¶^ ^c^ Limited information on vaccination and ANC awareness^¶c^	Empowerment community midwives ^ȸ^ Encouraging care-seeking behaviors^ȸ^ Early recognition of danger signs ^c^ Use of technology ^c^ Mobile clinics^ c^ Partnership and collaborations^ȸ^ Community outreach to strengthen via midwives^ȸ^
Refugee-centered care ^c^ Support groups ^c^ Flexibility in guidelines^c^ Guidelines in different languages^c^ Community outreach^ȸ^	Perception of new Ebola or COVID-19 guidelines^ ɸ^ ^ȸ^ Mobility restrictions ^§ c^ Communication media ^§ c^ No translator/community support ^ɸc^ New attendance policies restricted access^ɸc^	Antenatal care	Paucity of healthcare personnel ^¶ ȸ^ Insufficient resources ^¶ȸ^ Redistribution of resources ^¶^ Rigorous Ebola or COVID-19 protocols ^ɸȸ^ Culturally attuned care missing – limited availability of female staff ^ɸc^ Missed recommended antenatal visits ^ɸȸ^ Paucity of healthcare personnel^ȸ^ Inadequate infrastructure, lack of supplies as well as PPE^ȸ^ Lack of data accuracy^e^	Use of technology^ c^ Mobile phone applications ^c^ Strengthening partnerships ^ȸ^ Community outreach^ȸ^ Capacity development and training support and empowering health workforce^e^ Enhance rural health services^e^
Refugee-centered care^c^ Encouraging facility delivery^ȸ^ Community messaging ^ȸ^ Virtual family interactions^c^ Community outreach^ȸ^	Birth at home preferred/more trust on traditional practices at the time of pandemic¶^ȸ^ Lack of information on comprehensive services^§ ȸ^ Interaction with healthcare providers^§^ ^ȸ^ Apprehension of contracting Ebola or COVID-19 or Fear of potential COVID-19 exposure^§ ȸ^ Limited financial resources^§ ȸ^ Perceived risk of death due to Ebola—information on high case fatality^e^	Intrapartum care	Traditional birth attendants available ^¶ ȸ^ New/modified service delivery model^ɸ c^ Service disruptions ^¶ ȸ^ Government lockdown measures ^§ ȸ^ Culturally attuned care missing – limited availability of female staff ^ɸc^ Paucity of healthcare personnel^ȸ^ Inadequate infrastructure, lack of supplies as well as PPE^ȸ^ Lack of data accuracy^e^	An efficient transport system with 24/7 availability^c^ Limiting delays in accessing emergency obstetric care ^ȸ^ Increasing trust in health workforce ^ȸ^ Community outreach^ȸ^ Enhance rural health services^e^
Refugee-centered approach^c^ Mother-physician messaging ^c^ Virtual family meetings^c^ Community support persons ^c^ Community outreach^ȸ^	Perception of new Ebola or COVID-19 guidelines^ȸ^ Suspended language support^§^ Restrictive visitor policies^§^ Forcible mother–child separation^§c^ Home-delivered newborns rejected for hospital care^¶^ ^c^ Perceived risk of death due to Ebola—information on high case fatality^e^	Postnatal care	Restricted family or community support^§cc^ Hospital-imposed restrictions on visitors^ɸc^ Early discharge of postpartum women. ^ɸc^ Lack of assistance from trained healthcare professionals ^¶c^ Inadequate infrastructure, lack of supplies as well as PPE^ȸ^ Lack of data accuracy^e^	Strong partnership of health care providers and other MNCH stakeholders ^ȸ^ Equity-based approach for displaced populations ^c^ Enhance rural health services^e^
	Suspension of community outreach programs and campaigns. ^¶^	Vaccination	Limited childhood vaccination services. ^¶ȸ^ Added burden on routine staff^¶c^ Suspension of services^¶ȸ^ Surge of vaccine-preventable diseases ^¶ȸ^	Improvement in vaccination uptake ^ȸ^ Rollout of new vaccines ^c^
Interventions tailored to the needs of displaced populations. ^c^	Ebola and COVID-19 related restrictions ^§^ ^ȸ^ Low utilization of services ^¶ ȸ^	Childhood illnesses	Decrease in consultation visits ^¶^ ^c^ Virtual appointments decreased motivation^c^	
Interventions tailored to the needs of displaced populations. ^c^	Restrictions at facility ^ɸ ȸ^ Low service utilization^¶ȸ^ Lack of trust in health services or health workforce^e^ Perceived risk of death due to Ebola—information on high case fatality^e^	Family planning	Modified service delivery methods ^ɸc^ Transition to virtual provision ^ɸc^ Limited availability of long-term postpartum contraceptives ^ɸc^ Limited information for informed decisions ^ɸc^ Suspension of services^e^ Supplies shortage^e^ Lack of trust in health services or health workforce^e^	
Virtual communication Refugee-centered approaches. ^c^	Lack of communication medium and support^¶ c^	Nutrition	Restricted access to screening^¶ c^ Nutrition staff were reassigned^¶ c^ Reduced enrollment in feeding programs ^¶ c^	Mobile activity to identify children^c^
Community engagement, sensitization, and rebuilding trust^e^ Sensitization, and rebuilding trust^e^	Lack of trust in health services or health workforce^e^ Perceived risk of death due to Ebola—information on high case fatality^e^	Overall MNCH	Paucity of healthcare personnel^e^ Shortage of essential supplies^e^ Inadequate infrastructure, lack of supplies as well as PPE^e^ Lack of data accuracy^e^	Holistic Crisis Preparedness^e^ Capacity development and training support and empowering health workforce^e^ Resource availability for essential health services and supplies^e^ Enhance rural health services^e^

^¶^Emerged from the themes of studies conducted in LMICs or LICs. In of Ebola, all the studies reported here are conducted in LMICs or LICs

^ɸ^Emerged from the themes of studies conducted in HICs, mostly in case of studies conducted on refugees during COVID-19 pandemic

^§^Emerged from the themes of studies conducted in both LMICs or LICs and HICs

^c^Reported in the studies conducted in the context of COVID-19

^e^Reported in the studies conducted in the context of Ebola

^ȸ^Reported in the studies conducted in the context of Ebola as well as COVID-19

In some cases, the authors reported that suspension of routine vaccination services and diversion of human resources due to COVID-19 resulted in an outbreak of vaccine-preventable diseases, such as measles, among children under-five [48]. Borno State in Nigeria documented 1176 suspected measles cases, with 509 confirmed cases, in 2020 [48]. However, the authors did not report a drop in immunization coverage.

#### Health workforce constraints

‘Care during pregnancy and childbirth’ i.e., ANC and IPC were the domains in which most of the studies reported greater barriers [42, 43, 47, 49]. The most frequently described challenges faced by women were associated with health workforce constraints for both Ebola [50, 51], and COVID-19 [42, 43, 47, 49]. These constraints were mostly related to limited staffing, overburdened health services, or insensitive behavior of staff, which at times led women to prefer unskilled birth attendants [42, 47, 50, 52, 53]. In some settings the health system showed resilience despite huge financial and human resource constraints [54]. Issues also emerged around childhood-related nutritional interventions when the dedicated health workforce was diverted to other areas of COVID-19 care [42], as expressed by a multilateral organization worker: “*When the COVID-19 started… I would say nutrition really suffered, because… they are reassigning… all the nutrition staff to work on COVID-19 related activities. So, we noted that… all the upstream work that we do with policy guidelines… things were not moving, everything had to come to a standstill for like five months*” [42]. In LMICs, a number of women chose traditional birthing practices owing to service disruption [42, 55]. Similarly, routine immunization programs were significantly impacted during Ebola, primarily due to widespread disengagement of the population with healthcare services, but also due to a reduction in the outreach services provided by healthcare workers in the community [56]. The behavior of health workers was also a crucial element impacting continuity of care, as shared by one health worker from Liberia: *“We were told not to touch, so during Ebola, I did not treat. When someone brought a child, I did not know if they had Ebola or not, so I referred* [53] *.*

#### Funding limitations

Redirection of financial resources (e.g., hiring of new staff, equipment and medical supplies) to cover Ebola or COVID-19 needs is a challenge [42, 44, 52, 53] One of the staff members of an NGO mentioned:* “Before the pandemic for certain activities did not materialize and planned programs didn’t open. In Yemen we scaled back antenatal and delivery care in an area in the knowledge that another actor had received funding and would start delivering these services; however, in the end this actor was not able to open the expected services, citing funding issues…”* [42]

#### Security

The security threat to the healthcare workforce in areas affected by conflict is concerning. Pandemics and epidemics have imposed a double burden to this risk, as explained by health staff: *"It will also be good to increase remuneration. Yes, for healthcare workers who have been at the forefront of this…fight, because for one in a humanitarian crisis setting, the major challenge is their insecurity and security threats. In addition, we have COVID-19 to contend with. So, it’s double trouble for each and everyone us."* [57]

#### Gender sensitivity

Issues surrounding cultural sensitivity (e.g., limited access to female staff) have emerged as important challenges to care seeking [45, 58]. With regards to ‘community outreach’ programs, accessibility issues related to their suspension [42, 52], and to limited female workforce available to perform outreach visits because of the diversion of staff to cover pandemic-related care were reported [44, 45]. The suspension of FP services or delays in providing contraceptives has also been documented in the studies [42, 45].

### New mode of service delivery

**Fig. 2:**
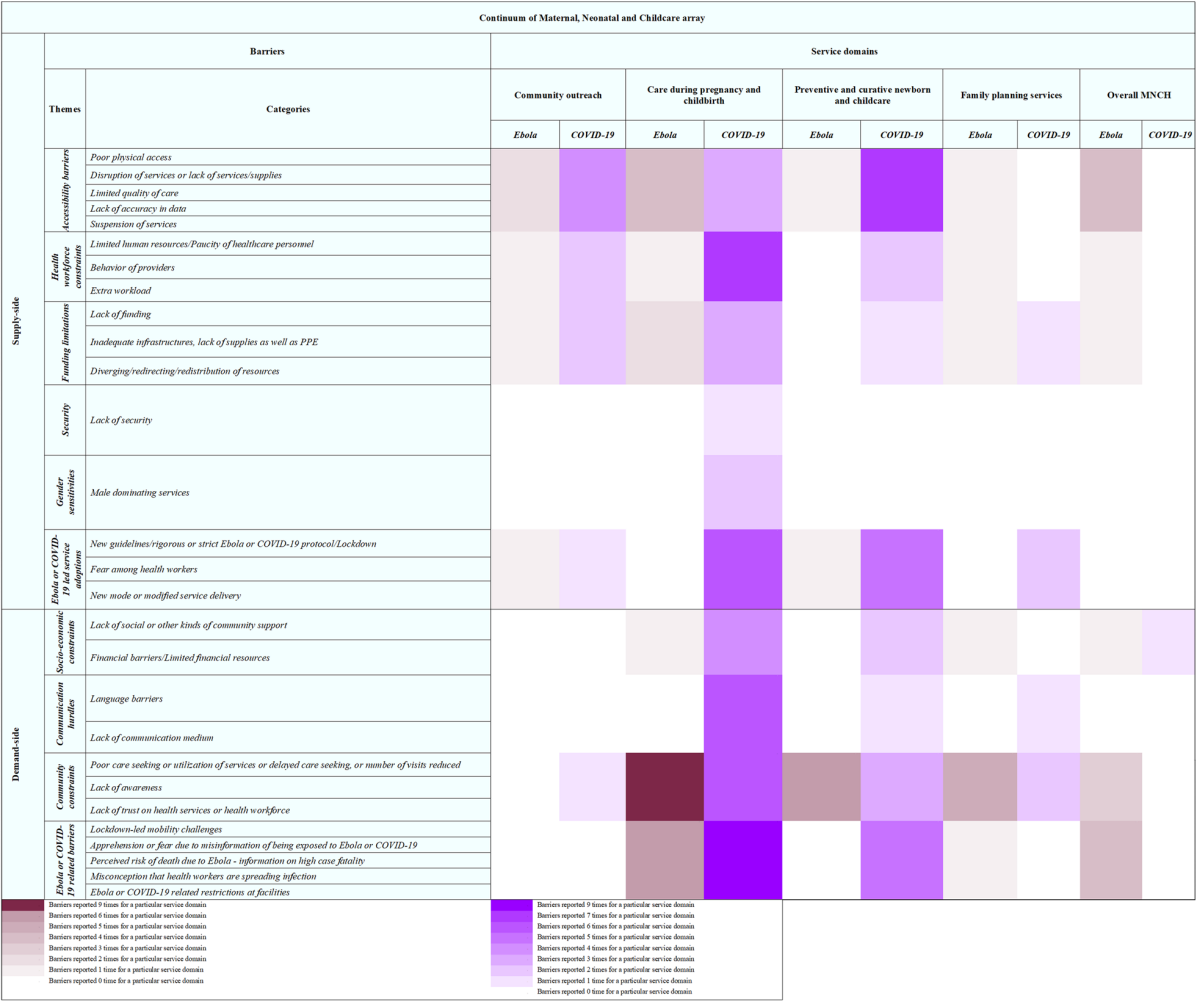
Common barriers impacting continuity of care. Thematic analysis of the findings of the included studies

Among refugees in HICs, new service delivery modes connected to the COVID-19 outbreak, such as mask restrictions, new policies on isolation protocols, and virtual appointments for antenatal and postnatal care, were the most frequently documented obstacles [44, 45]. Similarly, COVID-19 related restrictions in host HICs imposed harsh experiences at facilities, as shared by one woman: “*The hospital rules are strict during COVID. Visits are forbidden, friends can’t come, and they could not be there to help me.”* [45] The experiences of pregnant women during Ebola were even worse, and challenges were even more complex, especially because of the high case fatality rate in many West African countries [51, 59–61]. In HICs, the new guidelines imposed at health facilities after the start of the COVID-19 pandemic led to severe concerns among refugee women in need of PNC [44, 45]. One refugee woman called attention to her experience: *“When the COVID-19 pandemic hit, they [healthcare providers] gave us a certain time for visits, 2 h in the morning to see the baby [sick and hospitalized child] and to meet the doctor and 2 h in the night, and I was the only one allowed to visit”.* [44] Furthermore, services connected to PNC and child illnesses other than nutrition were also affected, and access to care was hampered as a consequence of the new modalities of service delivery and disruption of care provoked by the pandemic [43, 44]. Visitor restriction policies during childbirth negatively affected the mental state of many pregnant women, as reported by one study participant: “*No one could accompany me to the hospital because of COVID. I was alone. My husband drove me to the hospital, but I was all by myself during delivery. My husband helped me carry my things with me to the hospital, but other than that I was all by myself”* [45] (Fig. 2)*.*

### Challenges affecting continuity of care at demand-side

#### Socio-economic constraints

In the context of conflicts, the challenges of poverty and inaccessible healthcare are further exacerbated. During periods of conflict, government-provided maternity services are often unavailable or severely limited [52]. This creates a vacuum in healthcare that is only partially filled by private facilities [52, 62]. However, these private facilities, while operational, are frequently too expensive for most of the population. The situation is compounded during emergencies such as the COVID-19 pandemic, when the demand for healthcare services, particularly maternity care, surges dramatically [42]. With government facilities being either non-functional or overwhelming, many expectant mothers are left with no choice but to seek services from private hospitals. This high demand for limited supply in private facilities not only inflates costs but may also lead to overcrowded conditions, further diminishing the quality of care [42]. Moreover, in conflict zones, the physical and logistical challenges of reaching healthcare facilities become even more daunting [47]. The risks of traveling, combined with the disruption of usual transportation services, often result in many deliveries occurring at home, without professional medical assistance [52].

### Communication hurdles

“Communication hurdles” emerged as the main challenge for many refugee women living in HICs, who were concerned of suspension or modification of interpreter services [44, 45] and of the language barriers that they could encounter while seeking PNC and childcare in both HICs and LMICs [44, 45, 52]. With regards to community awareness, there was misinformation that led to disease spread, as shared by one health professional during Ebola: *“We were not prepared, by that time, we had no training and were not knowledgeable. The message that was around was that when you eat bats and mangoes, you will be infected. We believe that we will get infected through that. It is only during the intense phase [of the epidemic] that we came to know it was transmitted through body contact, sweat saliva, and things like that’* [63]*.* Linguistic barriers were one of the most frequently reported challenges identified in the domain of pregnancy care: *“Sometimes they explained things to me by using signs and I understand a little English, but it’s hard to understand medical terms and they didn’t use an interpreter for this — a refugee women”* [45]*.* One woman reported: “*If you cannot speak like in isiZulu here in KZN, they will just go on with their languages until you feel like you can go crazy. And then you start to answer things you do not even know. You are not expected to say I don’t understand isiZulu”* [64]*.*

### Community constraints

Some were also concerned about being discriminated against by health providers due to past experiences connected to ethnic discrimination, mistreatment, and refusal of care, as highlighted by a health provider: “*They [Rohingyas] fear being tortured at hospitals, or the doctors will not treat them correctly. They have this belief. This situation has worsened during the pandemic. We encountered many cases in which they refused to go to hospitals. They think they will be maltreated even more because of COVID [if suspected or diagnosed with it] …They believe that the doctors would not care for them as Rohingyas.”* [47] Mistrust of the existing health system in LMICs led refugee women to avoid care seeking, as shared by a community worker: *“It seemed like a lot of our clients would make sure they are very, very sick before they go to hospitals…. They completely avoid going. They say until I feel like I am about to die I won’t go, because I don’t want to face that system” *[64]*.* Additionally, care across MNCH domains was affected by constraints (e.g., poor care seeking behaviors, lack of awareness of health conditions, and lack of trust) [42, 47, 52, 57, 63, 65]. Low utilization of services was provoked by refugee women’s health status, lack of awareness of available services, and limited knowledge of facility guidelines during the pandemic [47, 55, 65]. Most women experienced these challenges while in need of neonatal and childcare services, ANC, and IPC [45, 51]. Misconceptions related to the spread of the disease and fear affected care seeking behaviors among women [69]. FP was also affected, as explained by one of the women who stopped using contraceptives during Ebola: “*Because during that time we were afraid, we felt they were giving Ebola [injections] or if you go to seek prevention, you might not know the person who is treating you, they might give you another injection that is not prevention, so that was why we were afraid during that time*.” [70]

### Ebola or COVID-19 related barriers

On demand-side, ‘Care during pregnancy and childbirth’ carried the greatest impact [42, 44, 45, 47, 52, 55, 66, 67]. During the pandemic, the lockdown was perceived with great fear, negatively influencing women’s care-seeking behaviors for ANC and IPC. During Ebola in Guinea, ANC attendance, PNC visits, and facility-based births decreased by 18%, 22%, and 11%, respectively. This decline led to a 34% increase in facility maternal mortality ratio and a 24% increase in stillbirths [68]. A woman shared the experience during Ebola: *“I decided to give birth at home during Ebola because they made the thing so fearful; that when you went [to the clinic] they will put you into a vehicle and then go and kill you”* [51]*. *Avoidance of access to care due to lockdown-related restrictions and due to fear of contracting Ebola and COVID-19 at facilities was also common in LMICs [52], as shared by a health professional: “*Before the outbreak of Corona, they were coming to the hospital in large numbers. They used to come, many of them, but now the numbers have reduced drastically because they fear coming to the facility.”* [52] Many pregnant women and mothers were hesitant to seek essential healthcare services, fearing being exposed to Ebola, especially given the close physical contact required during maternity care and the possibility of becoming infected in healthcare settings [63]. The high fatality rate led to an atmosphere of fear and panic, which deterred individuals from visiting health facilities, even when they needed critical medical care. This apprehension severely affected the utilization rates of MNCH services, leading to potentially preventable maternal and neonatal complications and deaths. A health professional working during Ebola shared his experience: *“‘During that time initially the community people feared to come to the health facility thinking that sometimes if they come, they will be infected with Ebola.”* [63] Moreover, women also perceived use of PPE during Ebola as one of the barriers to access care, as explain by one of the community women: *“That is why some are afraid to go to the center because of the PPE…because when they wear the PPE is like a ghost, even if you know someone, when they wear the PPE, you will not recognize the person.”* [69] Similarly, fear of contracting COVID-19 caused avoidance of care-seeking behaviors in LMICs, as explained by one health staff: “*Yes. We have a lot of home deliveries. In fact, we were discussing yesterday that home deliveries have gone up, and it has gone to an extent that even those mothers feared coming to the hospital*”. [51] Another refugee woman explained: *“I think COVID-19, changed life itself, especially during the lockdown times. We are not able to see doctors as easily because of [a] fear of going to the hospital”* [44]*.* (Fig. 2).

### Strategies to overcome supply-side challenges

**Fig. 3:**
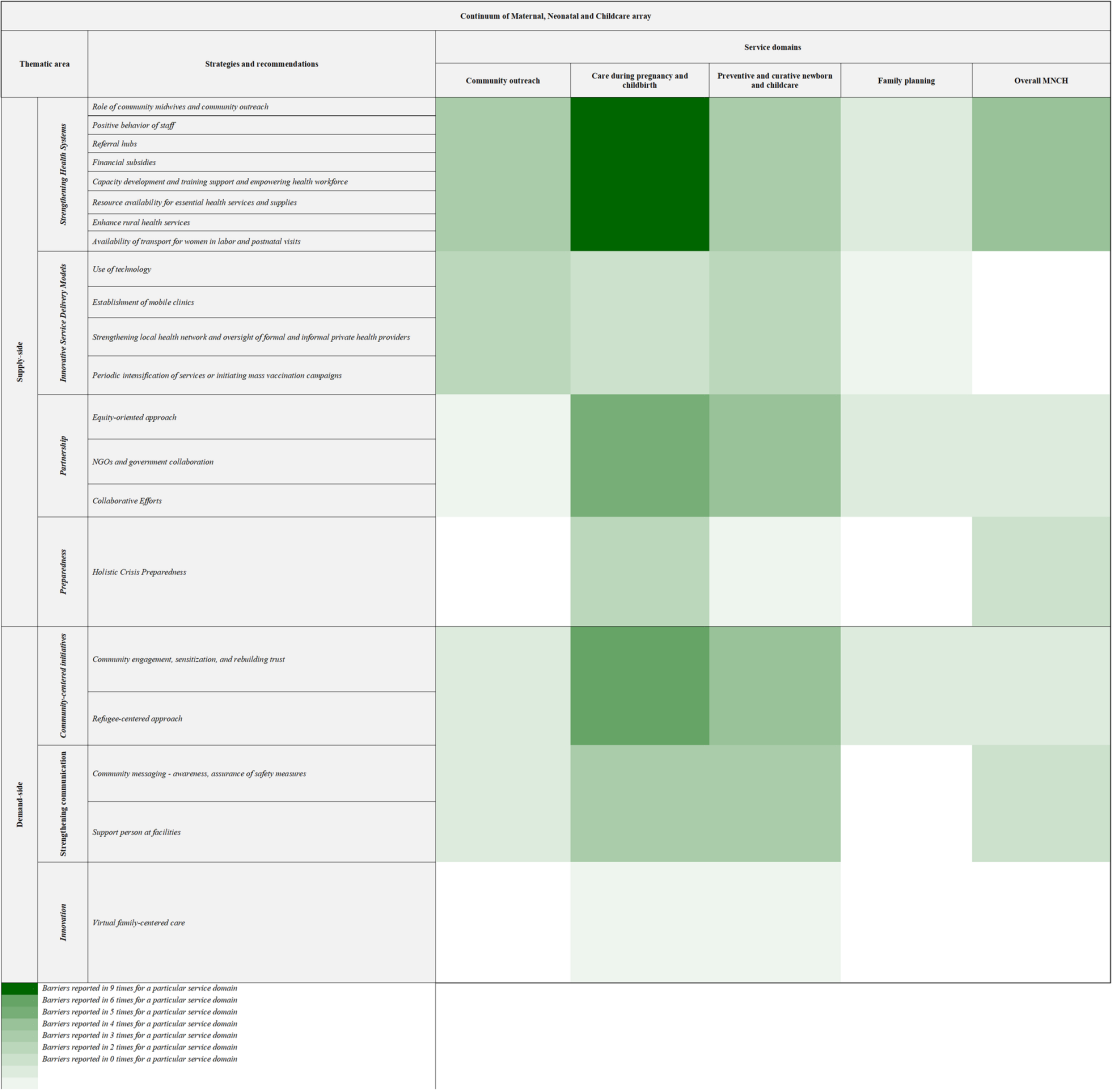
Strategies implemented or recommended in articles to ensure continuity of care. Driven by a thematic analysis of the content of the studies

Nine strategies were reported on the supply side. ‘Partnerships between different stakeholders’ were the most frequently mentioned ones across key MNCH domains [42, 45, 47, 52, 55, 64]. The studies highlighted that collaboration between government and humanitarian actors as well as communities was integral to the delivery of MNCH services. This is even more crucial in the context of LMICs, where countries are more strained by the dual impact [42, 52, 55]. Equity-oriented approaches aiming to keep refugees in the center of decision-making were identified as strategies that could potentially address the challenges faced by populations affected by conflict while seeking ANC and childbirth care in HICs [44, 45]. ‘Strengthening health system’ by improving referrals system and improving access to transport system could be used to improve access to care, as reported in one study conducted in Bangladesh for Rohingya refuges in Cox Bazar, where this was employed to support refugee women in accessing emergency obstetric care [47, 55]. Strategies that strengthen the role of midwives were also described as crucial in delivering better services [42, 47, 53]. Similarly, ‘innovation in service delivery,’ for example use of technology and mobile health application tailored according to the needs of the population accessing MNCH services, was also highlighted as a pathway for improving access to care [42, 45, 70]. The ‘intensification of services’ by rapid outreach for vaccination at community level during COVID-19 was identified as a key measure that could avert the loss in gains in service indicators due to pandemic-induced disruptions [48, 70]. (Fig. 3).

### Strategies to overcome demand-side challenges

Community-centered initiatives, such as community engagement and refugee-centered approaches, have been emphasized as leading ways to improve access across the continuum of care [45, 53, 57, 63]. Furthermore, ‘strengthening communication’ with women through community messaging and community support systems has been described as a key solution to achieving the optimal utilization of services [46, 49, 56]. and continuity of care. [58, 69] This pathway has been reinforced in several studies, especially for the care of pregnant women, preventive and curative care of newborns, and childcare [42, 44, 45, 52].

## Discussion

Among MNCH service domains, supply-side factors have been reported in more studies compared to demand-side factors [42, 44, 45, 49, 52, 53, 64, 65]. Care during pregnancy and childbirth, specifically IPC, had the highest number of reported challenges [42, 44, 45, 48, 52, 55, 66, 68, 70], while nutrition had the lowest [42].

The limited number of included studies confirms the data gap existing in research focusing on populations affected by armed conflicts and with the additional burden of pandemics or epidemics. These findings suggest that Ebola and COVID-19 led to a change in service provision both at supply-side (e.g., new guidelines or strict Ebola and COVID-19 protocols, restrictions, and new or modified service delivery mechanisms) and on the demand-side (e.g., restriction-led mobility challenges, apprehension, or fear of being exposed to Ebola or COVID-19).

In HICs, refugee women and children encountered challenges in accessing MNCH services due to cultural and linguistic barriers, lack of tailored healthcare services, and limited awareness of available resources [30, 71–73]. The findings of this review highlight that after the start of the pandemic many social and linguistic services previously available for refugee women in HICs were either suspended or postponed, hindering care seeking patterns in ANC, IPC, and PNC [44, 45]. These challenges were amplified after the implementation of the new guidelines, and restrictions affected the general lives of women and children, as well as their access to facilities. Refugees from LMICs sometimes have difficulties understanding the care provision in HICs and might also face financial constraints [30]. Therefore, displacement to a new location can compromise their ability to receive adequate care [30, 74], and this effect may be exacerbated by pandemics or epidemics. In LMICs, women and children, whether refugees or internally displaced or living in countries affected by conflict, face different challenges in accessing MNCH services [71, 75]. Limited healthcare infrastructure, personnel, and essential supplies hamper the ability of under-resourced healthcare systems to provide adequate MNCH care [76]. The findings of this review show that during Ebola and COVID-19, the response of health systems was not optimal to fill these gaps in LMICs. This was provoked by pre-existing inadequate funding for healthcare services, geographical inaccessibility, and higher disease burden that further impedes access to PHC for the general population [77–79], and creates huge constraints for displaced women and children in accessing MNCH care [42]. Other key aspects that were highlighted in the included studies, which are also essential for ensuring continuity of MNCH care for our target population, are cultural beliefs related to gender preferences for health workers, which influence healthcare-seeking behaviors among refugee populations in LMICs [47, 55, 77, 78].

Differences in the impact of Ebola and COVID-19 on MNCH services in conflict settings are profound. While both pandemics severely disrupted health systems globally, their regional and temporal focuses were distinct. Ebola, with its higher mortality rate and more immediate, visible impacts, has typically garnered significant attention and fear in these settings [80]. The visibility of Ebola's effects, coupled with its rapid transmission within communities, necessitated urgent healthcare interventions [81]. In contrast, COVID-19, despite its global significance, often did not receive the same level of immediate concern in conflict-affected regions [82]. This difference in perception can be largely attributed to the lower mortality rate of COVID-19, coupled with a lack of testing infrastructures leading to a high proportion of undetected asymptomatic cases [83]. As a result, in many conflict-stricken areas, the response to COVID-19 was subdued or delayed, overshadowed by more pressing health emergencies and the direct impacts of ongoing conflict [84]. Further, Ebola predominantly affected West African nations, where health systems were already strained due to prolonged conflicts and limited resources [13]. The challenges during the Ebola outbreak were intensified by community mistrust, limited health infrastructure, and the virulence of the disease itself, which at times caused healthcare providers to abandon health facilities. On the other hand, the COVID-19 pandemic, being a global crisis, stretched even well-resourced health systems in HICs [85], leading to wider service disruptions, including of MNCH services. The rapid spread of COVID-19 necessitated stringent restrictions and modifications in service provision guidelines, inadvertently widening the gap of healthcare access for vulnerable groups, especially in conflict zones. The contrast in regional concentration and global reach between the two pandemics has further highlighted the disparities and gaps in health systems’ preparedness and response, particularly in conflict settings.

Moreover, the strategies employed to address these health crises in conflict settings have varied significantly. The response to Ebola, for instance, often involved intensive community engagement and education, given the disease's high transmission rate and visibility [17, 86]. For COVID-19, the strategies were more complex, owing to its asymptomatic spread and the global challenge in managing the pandemic. However, there remains a gap in the literature regarding a comprehensive analysis of these strategies and their effectiveness in different conflict-affected settings. An in-depth discussion on how these strategies have been implemented, their successes, and limitations could provide valuable insights for health service providers in similar contexts. Such an analysis could focus on factors like resource allocation, community engagement, and the adaptation of public health measures under the constraints imposed by conflict. Understanding these strategies and their outcomes is essential for guiding future responses to health crises in similar challenging environments.

### Recommendations

The findings of this review strongly suggest that, it is pivotal that MNCH services remain culturally sensitive, with specific initiatives targeting these populations to grant continuity of care for women and children affected by conflict and facing the burden of global health emergencies [87]. We recommend that enhancing PHC is crucial to achieve universal health coverage (UHC) and for tracking progress towards achieving the SDGs, as it involves investing in accessible, well-equipped facilities and continued care [88–90]. One essential step in enhancing the availability and quality of MNCH services is to train healthcare workers on the specific needs of vulnerable pregnant women, mothers, and children under five and integrate essential MNCH services into PHC delivery [89, 91]. Moreover, to overcome geographical barriers and ensure that MNCH services reach refugees and IDPs living in conflict-affected countries in their settlements or homes, the implementation of mobile clinics or outreach services is vital [92]. To further strengthen these solutions, it is important to connect them with the World Health Organization (WHO) Health Emergency and Disaster Risk Management (Health-EDRM) framework [93]. This involves developing contingency plans and protocols specifically tailored to address the needs of vulnerable women and children affected by conflicts during global health emergencies [93]. This should also include equipping PHC facilities with necessary resources, establishing communication channels, and providing emergency response training for healthcare providers [93, 94]. Efforts to ensure continuity of care during and after emergencies should focus on providing access to essential services and medications, facilitating interpreter services, and coordinating with humanitarian organizations and communities [94]. This comprehensive approach is essential for providing better preparedness and response to the population affected by conflicts [89], and facing a double burden, such as vulnerable women and children. By addressing these barriers, countries can progress towards achieving UHC, which encompasses essential services for vulnerable populations affected by conflict, including refugees and IDPs [88]. Furthermore, prioritizing the well-being and empowerment of populations affected by conflict, refugee and IDP women, as well as of those who are living in conflict through access to maternal healthcare, including FP services and safe childbirth will help reducing gender disparities in health. [71, 95] Lastly, it is imperative to emphasize the role of a Disaster Risk Reduction for Resilience (DRR) approach in strengthening health systems in conflict-affected areas [96]. DRR, which focuses on pre-emptively building the resilience of communities and healthcare systems to withstand and recover from disasters, is particularly relevant in regions where ongoing conflict exacerbates the vulnerability to health emergencies [96]. Integrating DRR strategies involves not only preparing for immediate health crises but also building long-term resilience [97]. By embedding resilience-building measures into the healthcare system, such as reinforcing supply chains, diversifying healthcare delivery models (e.g., mobile clinics, telemedicine), and fostering community engagement in health planning and response, healthcare systems can be more responsive and sustainable in the face of both conflict and health emergencies [93].

### Strengths and limitations

The major strength of this review is that to our knowledge it is the first to analyze the existing literature on MNCH-related services for pregnant women, mothers, and children affected by conflict and facing a dual burden of to Ebola and Covid-19. Moreover, it is the first to summarize the barriers faced by these populations accessing MNCH and possible solutions on the demand- and supply- side. The limitations of the present review are connected to the small number of included studies the limited number of databases employed, as well as to the lack of inclusion of gray literature.

## Conclusion

In conclusion, the Ebola and COVID-19 emergencies have exacerbated the existing challenges in accessing MNCH services faced by people living in conflict, including refugees and IDP women and children coming from conflict-affected settings. Policymakers and stakeholders should acknowledge these issues and could take into consideration the potential solutions discussed in this study. By developing targeted interventions, policymakers can enhance the accessibility and quality of MNCH services for women and children living in conflict, refugees, and IDP women and children in diverse host countries. By addressing these issues, we can contribute to the realization of SGDs and create a more equitable and inclusive healthcare system for all.

### Supplementary Information

**Additional file 1: Table S1.**

**Additional file 2: Table S2.**

**Additional file 3: Table S3.**

## Data Availability

All the data used in the review is provided within the manuscript and supplementary materials.
