# Correction: Impact of Ebola and COVID‑19 on maternal, neonatal, and child health care among populations affected by conflicts: a scoping review exploring demand and supply‑side barriers and solutions

**DOI:** 10.1186/s13031-024-00576-7

**Published:** 2024-03-01

**Authors:** Yasir Shafiq, Elena Rubini, Zoha Zahid Fazal, Muhammad Murtaza Bukhari, Maheen Zakaria, Noor ul Huda Zeeshan, Ameer Muhammad, Luca Ragazzoni, Francesco Barone‑Adesi, Martina Valente

**Affiliations:** 1 CRIMEDIM – Center for Research and Training in Disaster Medicine, Humanitarian Aid, and Global Health, Università del Piemonte Orientale, Novara, Italy; 2 Department of Translational Medicine, Università del Piemonte Orientale, Novara, Italy; 3 Centre of Excellence for Trauma and Emergencies (CETE) & Community Health Science, The Aga Khan University, Karachi, Pakistan; 4 Harvard Humanitarian Initiative, Department of Global Health and Population, Harvard T.H. Chan School of Public Health, Bostan, USA; 5 Department of Pediatrics, Brigham and Women’s Hospital, Global Advancement of Infants and Mothers, Boston, USA; 6 Medical College, Aga Khan University, Karachi, Pakistan; 7 VITAL Pakistan Trust, Karachi, Pakistan; 8 Department for Sustainable Development and Ecological Transition, Università del Piemonte Orientale, Vercelli, Italy

**Correction: Conflict and Health (2024) 18:12**

10.1186/s13031-024-00572-x.

The Funding information section was missing from this article and should have read ‘ This work was supported by the work under the grant by Bill & Melinda Gates Foundation [Grant Number INV-061145].The funder has played no role in the drafting of the manuscript and the decision to submit for publication’.

The original article [1] has been revised.
