# Supplementary material for: Impact of Ebola and COVID-19 on maternal, neonatal, and child health care among populations affected by conflicts: a scoping review exploring demand and supply-side barriers and solutions
Source: Confl Health. 2024 Jan 30;18:12. doi: 10.1186/s13031-024-00572-x (PMC10829480; doi:10.1186/s13031-024-00572-x)
Supplement: Supplementary file 1 — Additional file 1: Table S1. [file 13031_2024_572_MOESM1_ESM.docx]

**Supplementary table 1 | Search strategy**

| ***Concept 1: Disease Outbreaks***  "Hemorrhagic Fever, Ebola"[Mesh] OR "Coronavirus Infections"[Mesh] OR "COVID-19"[Mesh] OR "SARS-CoV-2"[Mesh] OR “COVID-19 epidemiology”[Tiab] OR “COVID-19 outbreak”[Tiab] OR “COVID-19 pandemic”[Tiab] OR “SARS-CoV-2 pandemic”[Tiab] OR “EBV”[Tiab] OR Ebola[Tiab] OR [Tiab] |
| --- |
| "Disease Outbreaks"[Mesh] OR "Pandemics"[Mesh] OR "Epidemics"[Mesh] |
| ***Concept 2: Conflicts***  "Armed Conflicts"[Mesh] OR "Ethnic Violence"[Mesh] OR "Genocide"[Mesh] OR "Disasters"[Mesh] OR "Relief Work"[Mesh] OR "Emergencies"[Mesh] OR "Chemical Terrorism"[Mesh] OR "Terrorism"[Mesh] OR "Bioterrorism"[Mesh] OR “Civil Disorders"[Mesh] OR "War Exposure"[Mesh] OR humanitarian*[tiab] OR “Protracted armed conflict” [tiab] OR Conflict*[tiab] OR “conflict-affected settings”[tiab] OR Civil unrest [tiab] OR war [tiab] OR “conflict zone*” [tiab] “Conflict area*” [tiab] OR Persecution* [tiab] OR Hostilit* [tiab] OR Violence [tiab] OR War zone* [tiab] OR “Conflict-related”[tiab] OR “Civil unrest” [tiab] OR “War-torn region*” [tiab] OR post-conflict [tiab] OR “armed conflict”[tiab] |
| "Democratic Republic of the Congo"[Mesh] OR "Congo"[Mesh] OR "Guinea"[Mesh] OR "Liberia"[Mesh] OR "Afghan Campaign 2001"[Mesh] OR "Iraq War, 2003-2011"[Mesh] OR "Syria"[Mesh] OR Sierra Leone[Mesh] OR Mali[Mesh] OR South Sudan[Mesh] OR Sudan[Mesh] OR Uganda[Mesh] OR “Yemen”[MESH] OR “Somalia”[MESH] OR “Myanmar”[MESH] OR DRC[tiab] OR Rohingya[tiab] OR “Syrian refugee”[tiab] |
| "Refugee Camps"[MeSH] OR "Refugees"[MeSH] OR "Transients and Migrants"[MeSH] OR "migrant women"[tiab] OR "displacement*"[tiab] OR "internally displaced"[tiab] OR "internal displacement*"[tiab] OR "idp"[tiab] OR "refugee women"[ tiab] OR "conflict affected"[tiab] OR “conflict-affected population*”[tiab] OR “displaced famil*”[tiab] OR “crisis-affected”[tiab] OR “displaced mother*”[tiab] OR “refugee child*”[tiab] OR “Displaced famil*”[tiab] OR “conflict-induced displacement”[tiab] OR “refugee settlement*”[tiab] OR “conflict setting*”[tiab] OR resettled[tiab] OR “refugee camp”[tiab] OR FCAS[tiab] |
| *Concept 3: Maternal, Neonatal and Child Health*  "Maternal-Child Health Centers"[Mesh] OR "Pregnant Women"[Mesh] OR "Perinatal Care"[Mesh] OR "Prenatal Care"[Mesh] OR "Maternal Health*"[Mesh] OR "Maternal Health Services"[Mesh] OR "Child Health"[Mesh] OR "Child Health Services"[Mesh] OR "Infant Health"[Mesh] OR "Reproductive Health"[Mesh] OR "Reproductive Health Services"[Mesh] OR "Maternal-Child Health Services"[Mesh] OR "Vaccination"[Mesh] OR "Vaccination Coverage"[Mesh] OR "Immunization"[Mesh] OR "Maternal Mortality"[Mesh] OR "Child Mortality"[Mesh] OR "Infant Mortality"[Mesh] OR "Perinatal Mortality"[Mesh] OR "Perinatal Death"[Mesh] OR "Maternal Death"[Mesh] OR "Infant Death"[Mesh] OR "Pregnancy"[Mesh] OR "Family Planning Services"[Mesh] OR "Health Services Accessibility"[Mesh] OR "Newborn health"[tiab] OR “Health service”[tiab] OR “obstetric and newborn care”[tiab] OR “preventive measures”[tiab] OR fertility[tiab] OR mothering[tiab] OR “maternal, newborn, child health”[tiab] OR postnatal[tiab] OR “access to healthcare”[tiab] OR “utilisation”[tiab] OR “utilization”[tiab] OR nutrition[tiab] OR "cesarean section*"[Mesh] OR mothering[tiab] OR “obstetric service*”[tiab] OR “routine immunization”[tiab] OR “service utilization”[tiab] OR “Community-based”[tiab] OR “emergency obstetric”[tiab] OR "child"[Mesh] OR “referral*”[tiab] OR delay*[tiab] OR MNCH[tiab] OR MNCHN[tiab] OR “nutritional counseling”[tiab] OR “follow-up care”[tiab] OR “vaccination coverage”[tiab] OR “postnatal healthcare”[tiab] OR birth[tiab] ANC[tiab] OR “ante-natal care”[tiab] OR IPC[tiab] OR intrapartum[tiab] OR childbirth[tiab] OR “family planning”[tiab] OR “post-natal care”[tiab] OR “postnatal care”[tiab] OR RMNCH[tiab] OR “home deliver*”[tiab] OR antenatal[tiab] |
