# Supplementary material for: Impact of Ebola and COVID-19 on maternal, neonatal, and child health care among populations affected by conflicts: a scoping review exploring demand and supply-side barriers and solutions
Source: Confl Health. 2024 Jan 30;18:12. doi: 10.1186/s13031-024-00572-x (PMC10829480; doi:10.1186/s13031-024-00572-x)
Supplement: Supplementary file 2 — Additional file 2: Table S2. [file 13031_2024_572_MOESM2_ESM.docx]

**Supplementary table 2 | Inclusion and exclusion criteria**

| **Criteria** | **Inclusion** | **Exclusion** |
| --- | --- | --- |
| 1. **Language** | English | All other languages |
| 1. **Timeframe of publications** | 1990 and after  Rationale: Most trends at UNCHR on refugees are available from 1990 | Before 1990 |
| 1. **Type of studies** | Qualitative  Quantitative  Mixed methods  Case study  Field implementation reports | Policy brief  Perspective  Reviews  Letter to editor  Book reviews  Grey literature |
| 1. **Region** | All | - |
| 1. **Target population** | Women of reproductive age  Children under-five years  Neonates  Health providers | All other population groups like elderly |
| 1. **Context** | Conflicts: war, armed conflicts, persecution, ethnic violence, refugee camps, internal displacement, cross border conflicts  Countries labeled as fragile due to long civil war in the past  (WB/OCHA/UNHCR) | Natural disasters  Climate change |
| 1. **Pandemic** | COVID-19  Ebola | All other epidemics or disease outbreaks like Zika, Vaccine preventable diseases or small disease outbreaks like Measles, Malaria, Cholera, Polio |
| 1. **Type of health services** | All or any domain of MNCH-related health care services | Non-MNCH related services  Mental health only |
